# Short Report – Birth Weight is Not Associated With Cataracts or Pseudophakia – Results from the Gutenberg Health Study

**DOI:** 10.2147/OPTH.S502181

**Published:** 2025-01-16

**Authors:** Achim Fieß, Sandra Gißler, Stephanie Grabitz, Philipp S Wild, Karl J Lackner, Manfred E Beutel, Michael S Urschitz, Oliver Tüscher, Thomas Münzel, Jörn M Schattenberg, Stavros V Konstantinides, Norbert Pfeiffer, Alexander K Schuster

**Affiliations:** 1 Department of Ophthalmology, University Medical Center of the Johannes Gutenberg University Mainz, Mainz, Germany; 2 Center for Thrombosis and Hemostasis, University Medical Center Mainz, Mainz, Germany; 3 German Center for Cardiovascular Research (DZHK), Partner Site Rhine-Main, Mainz, Germany; 4 Preventive Cardiology and Preventive Medicine-Department of Cardiology, University Medical Center Mainz, Mainz, Germany; 5 Institute of Molecular Biology (IMB), Mainz, Germany; 6 Institute of Clinical Chemistry and Laboratory Medicine, University Medical Center of the Johannes Gutenberg-University Mainz, Mainz, Germany; 7 Department of Psychosomatic Medicine and Psychotherapy, University Medical Center of the Johannes Gutenberg University Mainz, Mainz, Germany; 8 Division of Pediatric Epidemiology, Institute for Medical Biostatistics, Epidemiology and Informatics, University Medical Center of the Johannes Gutenberg University Mainz, Mainz, Germany; 9 Clinic for Psychiatry and Psychotherapy, University Medical Center of the Johannes Gutenberg-University Mainz, Mainz, Germany; 10 Leibniz Institute for Resilience Research, Mainz, Germany; 11 Center for Cardiology-Cardiology I, University Medical Center of the Johannes Gutenberg-University Mainz, Mainz, Germany; 12 Department of Internal Medicine II, Saarland University Medical Center, Homburg, Germany; 13 Department of Internal Medicine I, University Medical Center, Mainz, Germany; 14 Department of Cardiology, Democritus University of Thrace, Alexandroupolis, 68100, Greece

**Keywords:** Cataract, pseudophakia, birth weight, epidemiology, population-based study

## Abstract

This study investigates the association between self-reported birth weight (BW) and the frequency of cataract and pseudophakia in a large population-based cohort in Germany, as part of the Gutenberg Health Study (GHS). Slit lamp examination and Scheimpflug imaging of 8205 participants, aged 35 to 74, were assessed and signs of cataract or pseudophakia analyzed. The research aimed to explore the correlation between fetal growth restriction and/or prematurity indicated by BW and the frequency of cataract and pseudophakia. In the univariable analysis, cataract was initially associated with low and high BW, but this association disappeared after adjusting for age, sex, examiner and cardiovascular risk factors. No association was found between low BW and pseudophakia or the frequency of cataract surgery within 5 years. The study reveals novel insights from a large population-based study specifically exploring this association.

## Introduction

The development of age-related cataract is a major cause of global visual impairment. Several risk factors have been discussed in cataract development, however research on the impact of perinatal factors, especially low birth weight (LBW) as a surrogate parameter of prematurity and/or fetal growth restriction on adult cataract development is scarce. The authors of a UK-based study suggest anatomical changes in the lens due to altered development in LBW children that may affect cataract genesis.^1^ In a previous analysis in our cohort, LBW correlated with lower visual acuity^2^ and altered ocular geometry,^3^ possibly indicating more severe lens opacifications. Adults born preterm may require earlier cataract surgery and face higher retinal complication risks.^4^ In another report of adults born extremely preterm, increased lens opacifications were described.^5^ Therefore, we aim to shed more light on LBW’s association with cataract and pseudophakia development as this is of high clinical relevance, aiding in early intervention and postoperative care.

## Materials and Methods

The Gutenberg Health Study (GHS) is an interdisciplinary, population-based, prospective cohort study (n=15,010 participants at baseline). In the present study, the presence of cataracts and pseudophakia was determined as part of a slit lamp examination at baseline. During a 5-year follow-up (5FU) examination, the lens status was assessed using Scheimpflug imaging.^6^ Self-reported birth weight (BW) was categorized as low (<2500g), normal (2500–4000g) and high (>4000g). Only participants with reported birth weight, slit lamp examination at baseline and sufficient Scheimpflug imaging were included in this study (n=8205). Logistic regression models with generalized estimating equations (GEE) were used to investigate the association of birth weight categories with the presence of cataract, pseudophakia at baseline and cataract surgery within a 5-year interval. The analyses were then adjusted for age, sex, and slit lamp examiner (model 1) and additionally for smoking, arterial hypertension, dyslipidemia, obesity, socioeconomic status, alcohol consumption, diabetes, glaucoma, and steroid medication (model 2).

## Results

In the study, 16410 eyes of 8205 adults were included (age 51.49 ± 10.62 years, 4386 women). Further participant characteristics are listed in Table 1. In univariable analysis, the results suggested 1.6-fold (p<0.001) higher odds for cataract development with LBW and 1.19-fold higher odds (p=0.05) with high birth weight (HBW). However, in model 1, after adjustment for age and sex, both effects did not remain significant (LBW OR 1.26, p=0.15; HBW OR 0.93, p=0.55). In model 2, adjusted for several confounders, birth weight also showed no significant association (LBW: OR 1.24, p=0.18; HBW: OR 0.9, p=0.37) at baseline. There was also no association between the presence of pseudophakia and BW, neither in univariable nor multivariable analysis (Model 2: continuous: OR 1.02, p=0.14; LBW: OR 0.91, p=0.77; HBW: OR 1.25, p=0.32) at baseline. When investigating the association of BW with the frequency of cataract surgery within 5 years, we also did not find significant associations in either model.

**Table 1:** Characteristics of the GHS Sample at Baseline (n=8205), Stratified by Study Groups

Birth weight	Overall	Group 1	Group 2	Group 3
	BW 1000–6000g	BW < 2500g	BW 2500–4000g	BW >4000g
Participants (n)	**8205**	**451**	**6727**	**1027**
Sex (Women) (%)	4386 (53.5)	304 (67.4)	3710 (55.2)	372 (36.2)
Age (y) ± SD	51.49 ± 10.62	53.43 ± 11.06	51.08 ± 10.52	53.30 ± 10.76
Birth weight (g) ± SD	3406 ± 656	1995 ± 390	3331 ± 401	4515 ± 420
SES ± SD	13.58 ± 4.30	13.09 ± 4.16	13.65 ± 4.28	13.40 ± 4.48
Obesity (yes) (%)	1943 (23.7)	115 (25.5)	1525 (22.7)	303 (29.5)
Body Mass Index (BMI, kg/m^2^) ± SD	27.08 ± 5.08	27.32 ± 5.93	26.90 ± 5.00	28.11 ± 5.06
Smoking (yes) (%)	1676 (20.4)	83 (18.4)	1359 (20.2)	234 (22.8)
Alcohol consumption, g/day ± SD	10.36 ± 15.72	9.02 ± 15.63	10.15 ± 15.43	12.34 ± 17.43
Steroid medication (yes) (%)	126 (1.6)	9 (2.0)	102 (1.5)	15 (1.5)
Arterial Hypertension (yes) (%)	3449 (42.1)	204 (45.2)	2763 (41.1)	482 (46.9)
Diabetes mellitus (combi-diagnosis) (%)	529 (6.4)	37 (8.2)	422 (6.3)	70 (6.8)
Dyslipidemia (yes) (%)	2455 (30.0)	148 (32.8)	1963 (29.2)	344 (33.6)
Glaucoma (ISGEO, yes) (%)	47 (0.7)	3 (0.8)	37 (0.7)	7 (0.8)
**Cataract parameters**				
Cataract (yes) OD (%) (BL)	1653 (20.1)	129 (28.6)	1295 (19.3)	229 (22.3)
Cataract (yes) OS (%) (BL)	1591 (19.4)	126 (27.9)	1241 (18.4)	224 (21.8)
Pseudophakia (yes) OD (BL / 5FU) (%)	222 (2.7) / 369 (5.5)	17 (3.8) / 32 (9.2)	164 (2.4) / 272 (5.2)	41 (4.0) / 65 (8.1)
Pseudophakia (yes) OS (BL / 5FU) (%)	215 (2.6) / 358 (5.6)	19 (4.2) / 34 (9.8)	159 (2.4) / 259 (5.0)	37 (3.6) / 65 (8.1)

**Abbreviations**: g, gram; mm, millimeter; mmHg, millimeter mercury; y, years; n, number; SES, socioeconomic status on a scale 3–12; BMI, Body Mass Index; kg, kilogram; m^2^, square meters; ISGEO, International Society of Geographical and Epidemiological Ophthalmology; OD, right eye; OS, left eye, BL, Baseline; 5FU, 5-year-Follow-up; SD, Standard Deviation.

## Discussion

In our cohort, LBW as a surrogate marker for prematurity and fetal growth restriction was not associated with an increased frequency of cataracts or pseudophakia in adulthood.

So far, studies assessing the effects of BW on the prevalence of cataract and the frequency of cataract operations have been scarce. A UK-based study from 2001 by Hall et al did not find consistent associations between size at birth and age-related cataract, when assessing this relation in logistic regression,^1^ which is in line with our results. Nevertheless, they found that nuclear cataract was associated with increased intrauterine growth, while other cataract types showed no significance. Their cohort, however, was about 20 years older than ours, which might be a crucial explanation to their results. In the Gutenberg Prematurity Study, the authors were able to find an increased lens opacification in participants born extremely preterm (≤28 weeks gestational age).^5^ Therefore, there may be an association of cataract and pseudophakia with prematurity rather than low birth weight, as these two factors cannot be used interchangeably. However, due to the missing knowledge of gestational age at birth of the participants in the GHS, we were unfortunately not able to assess this association. The results of the present study are further limited because the GHS included only a few participants with extremely low birth weight—a condition often linked to premature birth—and we may not have detected a significant association due to the lack of power. Furthermore, we may not have detected early lens changes due to a dependency of age and reported BW in our cohort, as well as ophthalmic examination in non-mydriatic eyes. We conclude that neither low nor high BW is associated with a higher presence of cataract or pseudophakia in participants aged 35 to 74 years. However, as previous reports have revealed a higher occurrence of ocular complications in individuals with LBW,^4^,^7^,^8^ BW is still a relevant parameter to be further assessed in the future, ie, using quantitative measures of lens opacities in an older cohort with less age variability.

## Data Availability

The analysis presents clinical data of a large-scale population-based cohort with ongoing follow-up examinations. This project constitutes a major scientific effort with high methodological standards and detailed guidelines for analysis and publication to ensure scientific analyses on the highest level. Therefore, data are not made available for the scientific community outside the established and controlled workflows and algorithms. To meet the general idea of verification and reproducibility of scientific findings, we offer access to data at the local database in accordance with the ethics vote upon request at any time. The GHS steering committee, which comprises a member of each involved department and the coordinating PI of the Gutenberg Health Study (PSW), convenes once a month. The steering committee decides on internal and external access of researchers and use of the data and biomaterials based on a research proposal to be supplied by the researcher. Interested researchers make their requests to the coordinating PI of the Gutenberg Health Study (Philipp S. Wild; philipp.wild@unimedizin-mainz.de). More detailed contact information is available at the homepage of the GHS (www.gutenberghealthstudy.org).
